# Comparison of lateral entry and crossed entry pinning for pediatric supracondylar humeral fractures: a meta-analysis of randomized controlled trials

**DOI:** 10.1186/s13018-021-02505-3

**Published:** 2021-06-09

**Authors:** Huaguo Zhao, Song Xu, Guanyi Liu, Jingyu Zhao, Shandong Wu, Linrui Peng

**Affiliations:** 1 Department of Orthopedics, Ningbo No. 6 Hospital, 1059 Zhongsandong Road, Ningbo, Zhejiang 315040 People’s Republic of China; 2 Department of Hepatobiliary Surgery, Shangyu People’s Hospital of Shaoxing, 517 Citizen’s Avenue, Shangyu, Shaoxing, Zhejiang 312300 People’s Republic of China; 3 Department of Orthopedics, Ninghai Hospital of Traditional Chinese Medicine, 1299 Taoyuan North Road, Ninghai, Ningbo, Zhejiang 315600 People’s Republic of China

**Keywords:** Supracondylar humeral fractures, Pediatric, Pin fixation

## Abstract

**Background:**

Closed reduction and pinning entry fixation have been proposed as treatment strategies for displaced supracondylar humeral fractures (SCHFs) in children. However, controversy exists regarding the selection of the appropriate procedure. Hence, this meta-analysis was conducted to compare the effect of lateral and crossed pin fixation for pediatric SCHFs, providing a reference for clinical treatment.

**Methods:**

Online databases were systematically searched for randomized controlled trials (RCTs) comparing lateral pinning entry and crossed pinning entry for children with SCHFs. The primary endpoints were iatrogenic ulnar nerve injuries, complications, and radiographic and functional outcomes.

**Results:**

Our results showed that iatrogenic ulnar nerve injuries occurred more commonly in the crossed pinning entry group than in the lateral pinning entry group (RR = 4.41, 95% CI 1.97–9.86, P < 0.05). However, its risk between the crossed pinning with mini-open incisions group and the lateral pinning entry group was not significantly different (RR = 1.58, 95% CI 0.008–29.57, P = 0.76). The loss of reduction risk was higher in the lateral pinning entry group than in the crossed pinning entry group (RR = 0.66; 95% CI 0.49–0.89, P < 0.05). There were no significant differences in the carry angle, Baumann angle, Flynn scores, infections, and other complications between these two groups.

**Conclusions:**

The crossed pinning entry with mini-open incision technique reduced the loss of reduction risk, and the risk of iatrogenic ulnar nerve injury was lower than in the lateral pinning entry group. The crossed pinning entry with mini-open incision technique is an effective therapeutic strategy for managing displaced supracondylar humeral fractures in children.

## Introduction

Supracondylar humeral fractures (SCHFs) are the most common type of elbow fractures in the pediatric population between 5 and 8 years old [1]. Numerous studies have reported that SCHFs occur with nearly equal frequency among females and males [2], accounting for approximately 10% of all fractures in children [3] and 70% of all pediatric elbow injuries [4]. Children are susceptible to this fracture, due to the bending function of the elbow, the weak metaphyseal sclerotin of the distal humerus, and the thin ridge of the metaphyseal bone between the coronoid fossa and the olecranon fossa. It has been reported that more than 95% of all SCHFs are extension-type injuries that occur during falls on an outstretched hand [5, 6]. At the same time, it is a troublesome injury with complications including neurovascular injuries, elbow stiffness, fascial compartment syndrome, malunion, and, especially, elbow varus deformities [7]. An SCHF has a great impact on the function and appearance of the elbow joint in children [8–10]. Extension-type injuries are classified according to Gartland’s criteria as type I (non-displaced and stable), type II (hinged fractures with intact posterior cortex), and type III (completely displaced) [11]. In 2006, Leitch et al. [12] added type IV, which identifies fractures with multidirectional instability. Complications, such as nerve palsies and loss of fracture reduction, could be found in types II and III [13].

Closed reduction and internal fixation using percutaneous pinning are the main treatments for SCHF. However, there are still some debates regarding the choice of pinning configuration for fixating the fractures. Currently, crossed pinning or lateral pinning using two or three pins is the most common pinning configuration for SCHF, although many reports have compared these two methods in terms of surgical outcomes, which one method produces the best functional outcomes remains controversial [14–16]. The two key factors for comparing the functional outcomes of the methods are elbow stability and the potential risk of iatrogenic ulnar nerve injury [17]. Medial/lateral crossed pinning fixation was reported to have better mechanical stability than lateral fixation [18]. However, iatrogenic injury of the ulnar nerve after medial pinning is a potential complication. Although several meta-analyses of medial/lateral crossed pinning versus lateral pinning for SCHF have been reported [17, 19–22], the conclusions drawn were based on the results from nonrandomized controlled trials (nRCTs), increasing the likelihood of biases. Randomized controlled trials (RCTs) are considered the most reliable form of scientific evidence in the hierarchy of evidence because they reduce the spurious inferences of causality and bias. Numerous RCTs have been published on this topic, providing the opportunity to perform a meta-analysis of the RCTs comparing lateral entry pin fixation with crossed medial and lateral entry pin fixation of displaced supracondylar fractures of the humerus in children.

As a powerful tool, a meta-analysis could provide more reliable results than a single study by combining all eligible studies, especially in explaining controversial conclusions. Moreover, randomized controlled trials (RCTs) had the highest evidence level. The current study was aimed to conduct a meta-analysis of RCTs to analyze the effect of lateral pin fixation and medial/lateral crossed pin fixation on iatrogenic injuries, functional outcomes, and complications in children with SCHFs, providing a reference for clinical treatment.

## Methods

### Literature search

We prospectively registered the protocol for this meta-analysis in the Preferred Reporting Items for Systematic Reviews and Meta-Analyses (PRISMA) international prospective register of systematic reviews (CRD42018095577). We conducted a literature search (up to July 2020) of the Cochrane Library, PubMed, EMBASE, Web of Science, the Chinese Biomedical Literature Database, the China Journal Full-text Database, and the VIP Database for studies comparing the effect of lateral pinning entry and medial/lateral crossover pinning entry in children with SCHFs. Additionally, we searched the reference lists of all the included publications and relevant reviews. Only articles published in English or Chinese were considered. The following main keywords were used: supracondylar fracture, humerus or humeral, pin or Kirschner wire, and randomized controlled trial or controlled clinical trial.

### Study selection

The inclusion criteria were (1) RCTs comparing lateral entry pinning with crossed entry pinning fixation for displaced SCHF, including Gartland types II and III; (2) patient age ranges between 1 and 15 years old; (3) displaced SCHFs, including Gartland types II and III; and (4) patients treated with closed reduction or mini-open incision. The exclusion criteria were (1) supracondylar fractures with proximal fractures and shaft fractures, (2) pathological fractures, and (3) observational and retrospective studies, case reports, cadaver or model studies, and biomechanical studies.

Two orthopedic reviewers independently scanned the titles and article abstracts and then reviewed the full text of the eligible articles after reviewing the abstracts. Disagreements were resolved by consensus discussion or by consulting with a third senior pediatric orthopedics. The methodological quality of the included studies was independently assessed by two reviewers. The quality was assessed using the following criteria described in the Cochrane Handbook for Systematic Reviews of Interventions: sequence generation, allocation concealment, blinding, incomplete outcome data, selective outcome reporting, and other sources of bias. A judgment of “yes” indicated a low risk of bias, “no” indicated a high risk of bias, and “unclear” indicated an unclear or unknown risk of bias.

### Data extraction

Two reviewers independently extracted the data from the included studies. Our data finally included the authors, the year of publication, study design, sample size, age, gender, the type of fracture, surgical method, the length of follow-up, and clinical outcomes. The clinical outcomes included iatrogenic ulnar nerve injury, radiographic outcomes (loss of reduction, carry angle, Baumann angle, change in Baumann angle [23], loss of carrying angle, and loss of reduction), functional outcomes (range of motion (ROM) described by Flynn et al. [24], return to full function, loss of elbow flexion, and loss of elbow extension), and complications (pin tract infections, superficial infections, and reoperations). The Baumann angle was assessed according to the criteria reported by Skaggs et al. [23] as follows: no displacement was defined as a change in the Baumann angle of less than 6°, mild displacement was a change of 6 to 12°, and major displacement was defined as a change greater than 12°. The functional results were graded according to the criteria of Flynn et al. [24], which are based on the carrying angle and elbow motion.

### Data analysis

The relative risks (RR) with 95% confidence intervals (95% CI) were calculated for dichotomous data, and the mean difference (MD) with 95% CI was calculated for continuous data. Heterogeneity between different studies was assessed by the χ^2^ test (significance level of *P* < 0.10) and the *I*^2^ statistic (*I*^2^ > 50% indicating significant heterogeneity). The results were pooled using the fixed-effects model for values of P > 0.10 and *I*^2^ < 50% or the random-effects model for values of P < 0.10 and *I*^2^ > 50%. Publication bias was assessed by Begg’s test and Egger’s test [25]. Statistical analyses were performed using RevMan version 5.3 (the Nordic Cochrane Center, the Cochrane Collaboration, Copenhagen, Denmark).

### Results

#### Description of studies

**Fig. 1:**
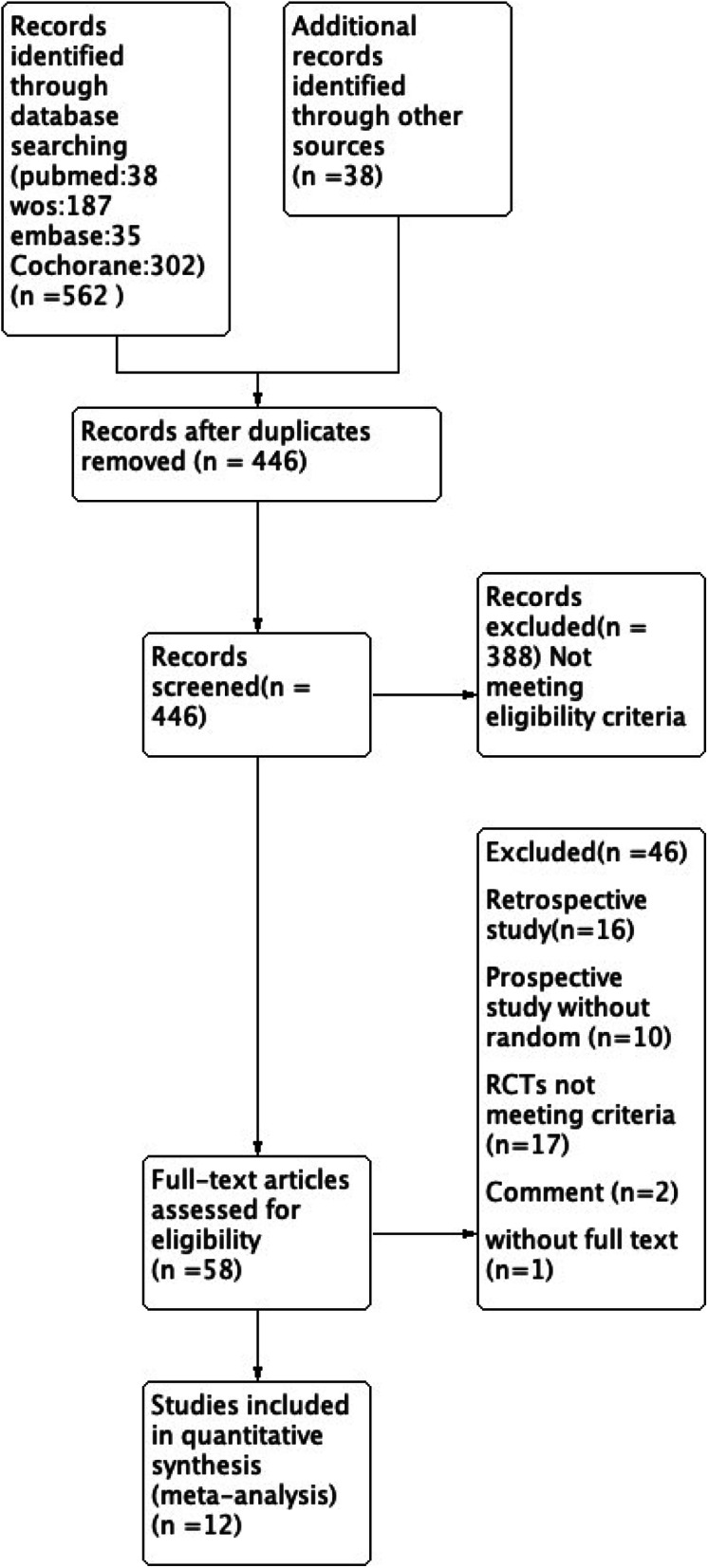
Flowchart of study search and inclusion criteria

**Fig. 2:**
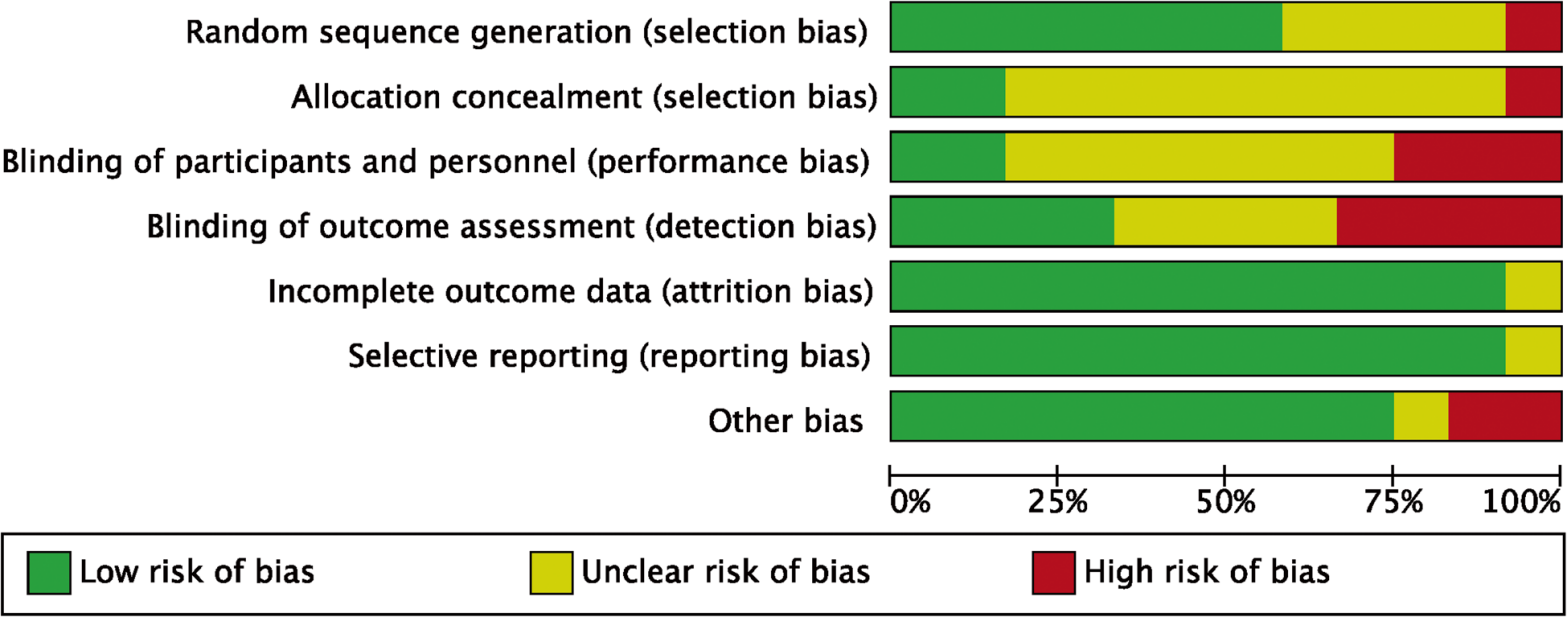
Risk of bias graph. The risk of bias item assessment across all included studies is presented as a percentage

**Fig. 3:**
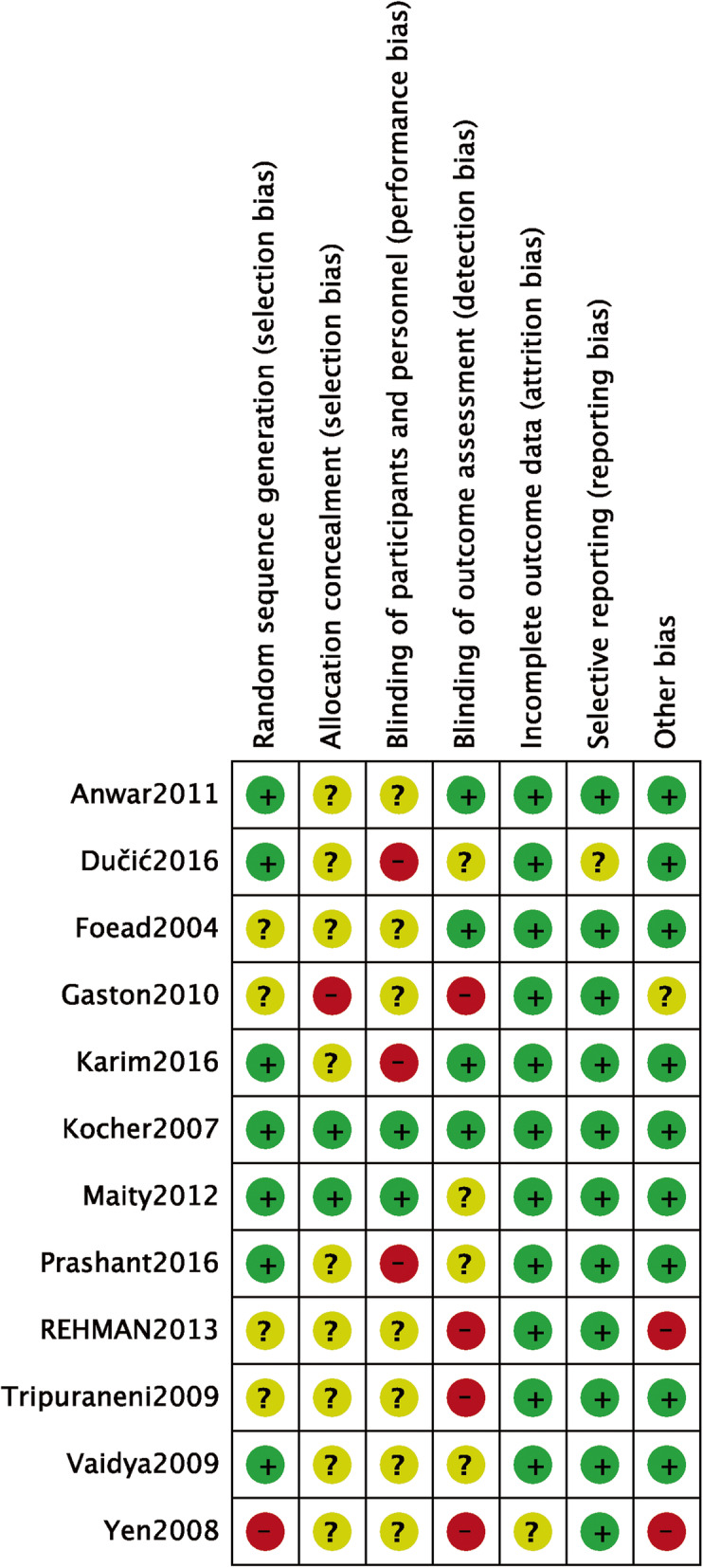
Risk of bias in the included randomized controlled trials. +, no bias; −, bias; ?, bias unknown

A total of 600 citations were obtained from the databases. One hundred fifty-four articles were excluded because of duplication, and 434 articles were excluded according to the inclusion and exclusion criteria. Finally, 12 RCT studies were included in the meta-analysis. The flowchart of the literature screening process is presented in Fig. 1. A total of 933 SCHF children (421 treated with crossed pins and 512 treated with lateral pins) were included, and the follow-up period ranged from 7.8 weeks to 36 months (Tables 1 and 2). The methodological quality assessment results were summarized in Figs. 2 and 3. All studies were described as randomized, but the method of generating the allocation sequence was not described in any article.

**Table 1:** Clinical characteristics of the included studies

Study	Year	Study design	Simple size		Mean age (years)		Gender (M/F)		Fracture type		Follow-up (months)	
			La	Cro	La	Cro	La	Cro	La	Cro	La	Cro
Ducic et al. [1]	2016	RCT	71	67	6.50 ± 1.85	6.70 ± 1.6	43/24	45/26	II/III	II/III	11.2 ± 2.3 (8.9–13.5)	11.2 ± 2.3 (8.9–13.5)
Prashant et al. [2]	2016	RCT	31	31	8.25	8.55	23/8	22/8	III	III	7.62 (32.64 weeks)	7.96 (34.12 weeks)
Shafi et al. [3]	2013	RCT	100	100	5.83 ± 1.83	6.25 ± 2.26	78/22	80/20	NA	NA	3	3
Maity et al. [4]	2012	RCT	80	80	6.12 ± 6.82	6.24 ± 1.77	64/16	48/32	II/III	II/III	36	36
Anwar et al. [5]	2011	RCT	25	25	7.02 ± 1.25	7.02 ± 1.25	NA	NA	II/III	II/III	6	6
Gaston et al. [6]	2010	RCT	47	57	5.70	6.20	22/25	31/26	III	III	NA	NA
Vaidya et al. [7]	2009	RCT	29	31	5.80	6.20	21/8	17/14	III	III	6.2	6.6
Tripuraneni et al. [8]	2009	RCT	20	20	4.30	5.50	NA	NA	II/III	II/III	2.17 (9.3 weeks)	1.82 (7.8 weeks)
Yen et al. [9]	2008	RCT	28	24	NA	NA	NA	NA	NA	NA	NA	NA
Kocher et al. [10]	2007	RCT	28	24	6.10 ± 1.50	5.70 ± 1.60	10/18	13/11	III	III	3	3
Foead et al. [11]	2004	RCT	32	34	5.78	5.78	NA	NA	II/III	II/III	NA	NA
Karim et al. [12]	2016	RCT	20	20	NA	NA	NA	NA	NA	NA	NA	NA

*La* lateral pinning entry, *Cro* crossover pinning entry, *M* male, *F* female, *II* Gartland type II fracture, *III* Gartland type III fracture

**Table 2:** Detail characteristic of included studies

Analysis item	Studies	Patients	Heterogeneity		Statistical method	Effect estimate	P value
			*I*^2^	*p*			
**Radiographic outcomes**							
Carrying angle	2	182	0%	0.95	MD (IV, random, 95% CI)	−0.08 (−0.90, 0.73)	0.85
Loss of carrying angle	4	297	0%	0.97	MD (IV, fixed, 95% CI)	−0.17 (−0.72, 0.38)	0.55
Baumann angle	2	182	0%	0.63	MD (M-H, fixed, 95% CI)	1.10 (−0.20, 2.40)	0.10
Change of Baumann angle	5	349	0%	0.86	MD (IV, random, 95% CI)	0.14 (−0.27, 0.54)	0.51
**Functional outcomes**							
Criteria of Flynn, excellent	7	792	0%	0.81	RR (IV, random, 95% CI)	1.05 (0.97, 1.14)	0.34
Criteria of Flynn, good	6	592	0%	0.92	RR (IV, random, 95% CI)	0.92 (0.60, 1.41)	0.71
Full return to function	2	112	0%	0.48	RR (M-H, fixed, 95% CI)	1.00 (0.91, 1.09)	0.96
Loss of elbow flexion	3	337	0%	0.91	MD (IV, random, 95% CI)	−0.18 (−1.65, 1.29)	0.81
Loss of elbow extension	3	335	0%	0.91	MD (IV, random, 95% CI)	−0.04 (−0.01, 0.09)	0.12
**Complications**							
Pin tract infection	4	337	0%	0.66	RR (M-H, fixed, 95% CI)	0.89 (0.37, 2.14)	0.80
Superficial infection	4	337	0%	0.58	RR (M-H, fixed, 95% CI)	0.87 (0.31, 2.44)	0.80
Reoperation	2	112	NA	NA	RR (M-H, fixed, 95% CI)	6.56 (0.35, 121.80)	0.21

Mild or major displacement (change of the Baumann angle) based on the criteria of Skaggs et al. [49]; excellent grading of criteria of Flynn et al. [13]

*MD* mean difference, *RR* risk ratio, *IV* inverse variance, *M-H* Mantel-Haenszel, *fixed* fixed effect, *random* random effect, *CI* confidence interval

#### Effects of interventions

##### Iatrogenic ulnar nerve injury

**Fig. 4:**
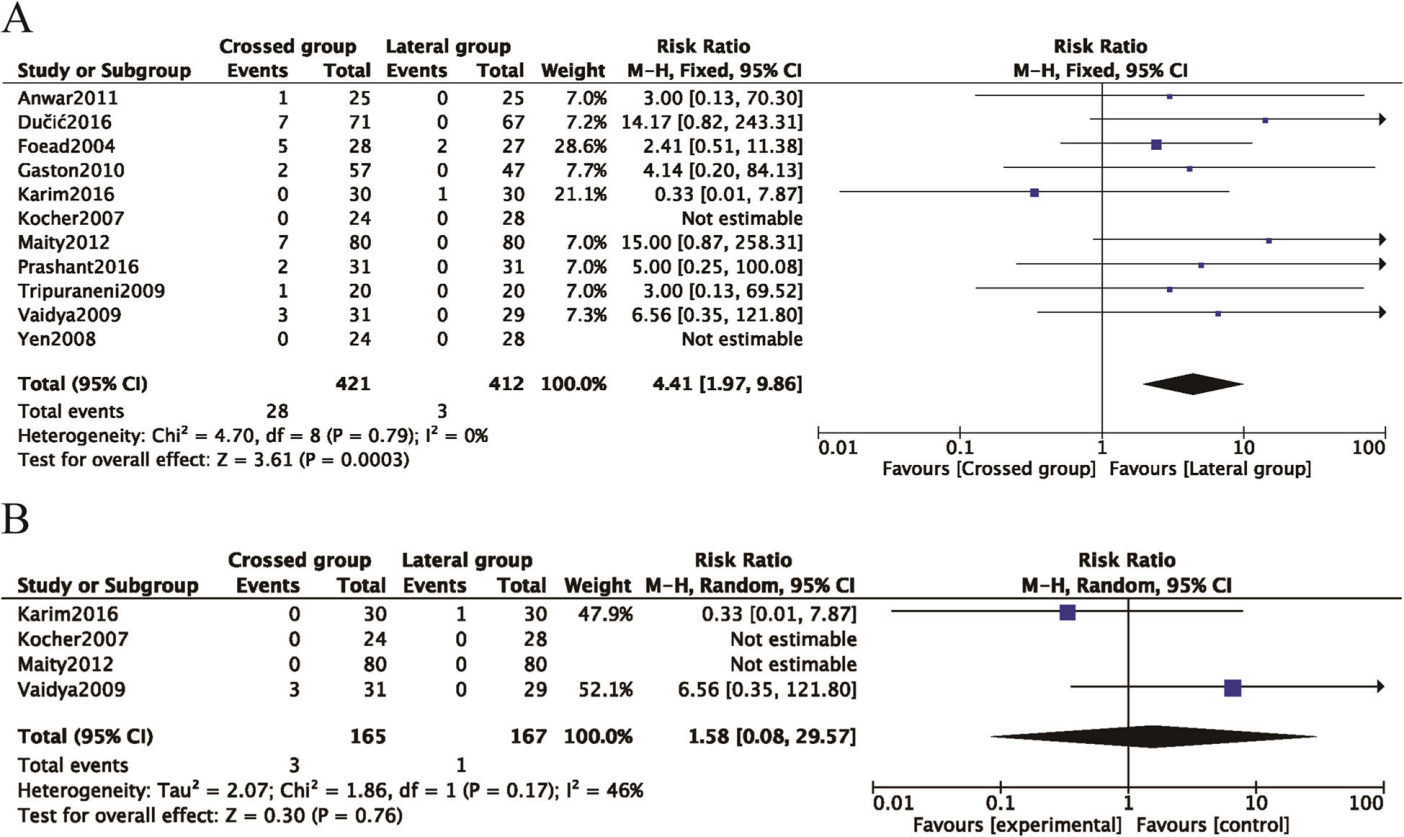
Comparison of iatrogenic ulnar nerve injury between the lateral entry group and the crossed entry group. **A** Iatrogenic ulnar nerve injury between the lateral entry group and the crossed entry group. **B** Iatrogenic ulnar nerve injury between the lateral entry group and the crossed entry group with a mini-open incision

**Fig. 5:**
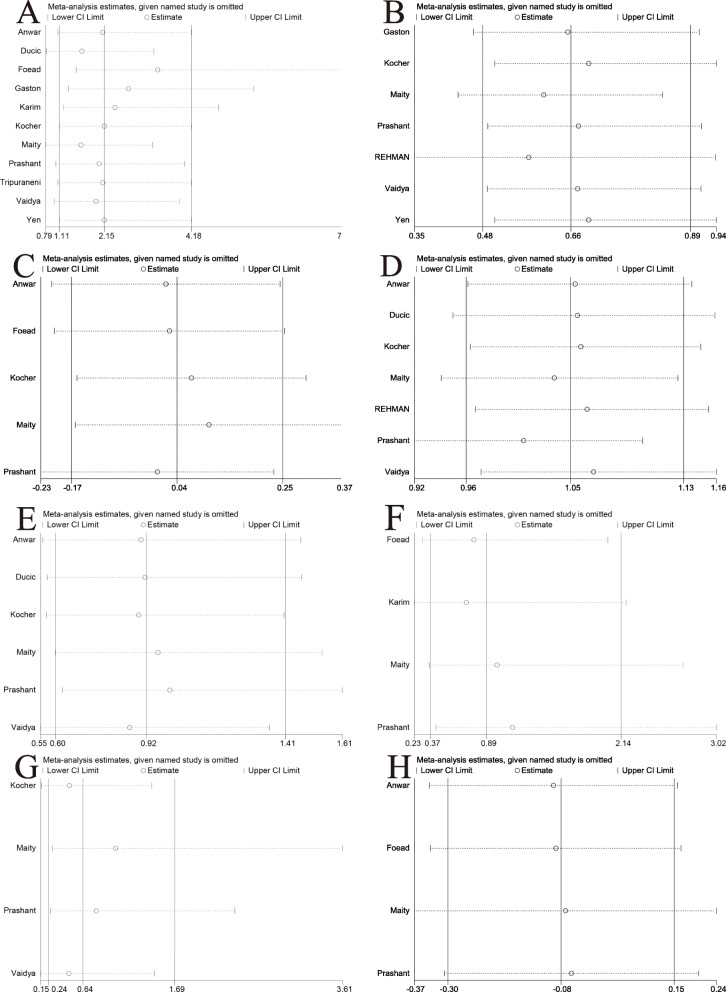
Sensitivity analysis of each included study. **A** Iatrogenic ulnar nerve injury. **B** Loss of reduction. **C** Change in Baumann angle. **D** Excellent Flynn criteria scores. **E** Good Flynn criteria scores. **F** Pin tract infection. **G** Superficial infection. **H** Loss of carrying angle

**Fig. 6:**
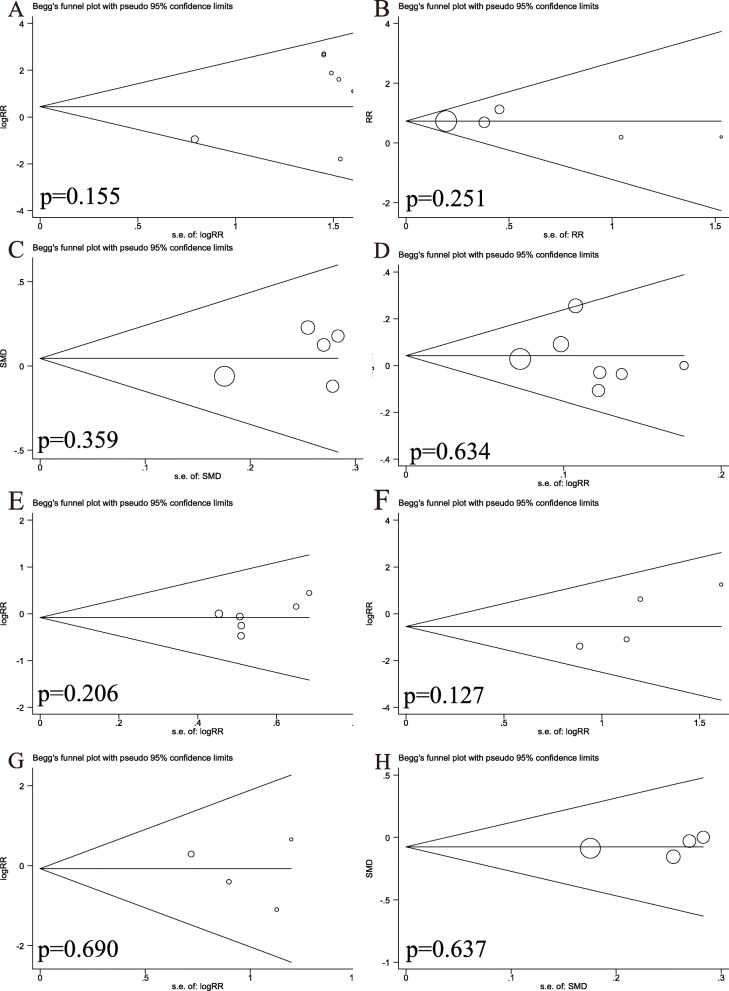
Begg’s publication bias plot. **A** Iatrogenic ulnar nerve injury. **B** Loss of reduction. **C** Change in Baumann angle. **D** Excellent Flynn criteria. **E** Good Flynn criteria. **F** Pin tract infections. **G** Superficial infections. **H** Loss of carrying angle

Iatrogenic ulnar nerve injury was one of the most common problems, reported in 11 of the 12 studies [8, 26–34]. The data were pooled across 11 studies, and the analysis revealed a significant difference in the pooled treatment effect with no heterogeneity (P = 0.79, *I*^2^ = 0%). Iatrogenic ulnar nerve injuries occurred more commonly in children treated with crossed pinning entry than in children treated with lateral pinning entry (RR = 4.41, 95% CI 1.97–9.86, P < 0.05) (Fig. 4A). Iatrogenic injury occurred in 28 (6.65%) of 421 children with crossed pinning and three (0.73%) of 412 children with lateral pinning entry. Publication bias was not significant (P = 0.155) (Figs. 5A and 6A).

In four studies in which children with SCHF were treated with crossed pinning with a mini-open incision [8, 26, 31, 33], the risk of iatrogenic ulnar nerve injury between the children treated with lateral pinning entry and the children treated with crossed pinning with mini-open incisions was not different (RR = 1.58, 95% CI 0.008–29.57, P = 0.76) (Fig. 4B). Iatrogenic injuries occurred in three (1.82%) of 165 children with medial and lateral pinning entry and one (0.60%) of 167 children with lateral pinning entry. The analysis of heterogeneity in this meta-analysis showed acceptable heterogeneity (P = 0.17, *I*^2^ = 46%). Due to the limited number of included studies (n  =   4), the bias funnel plot was not used for publication bias analysis.

##### Radiographic outcomes

**Fig. 7:**
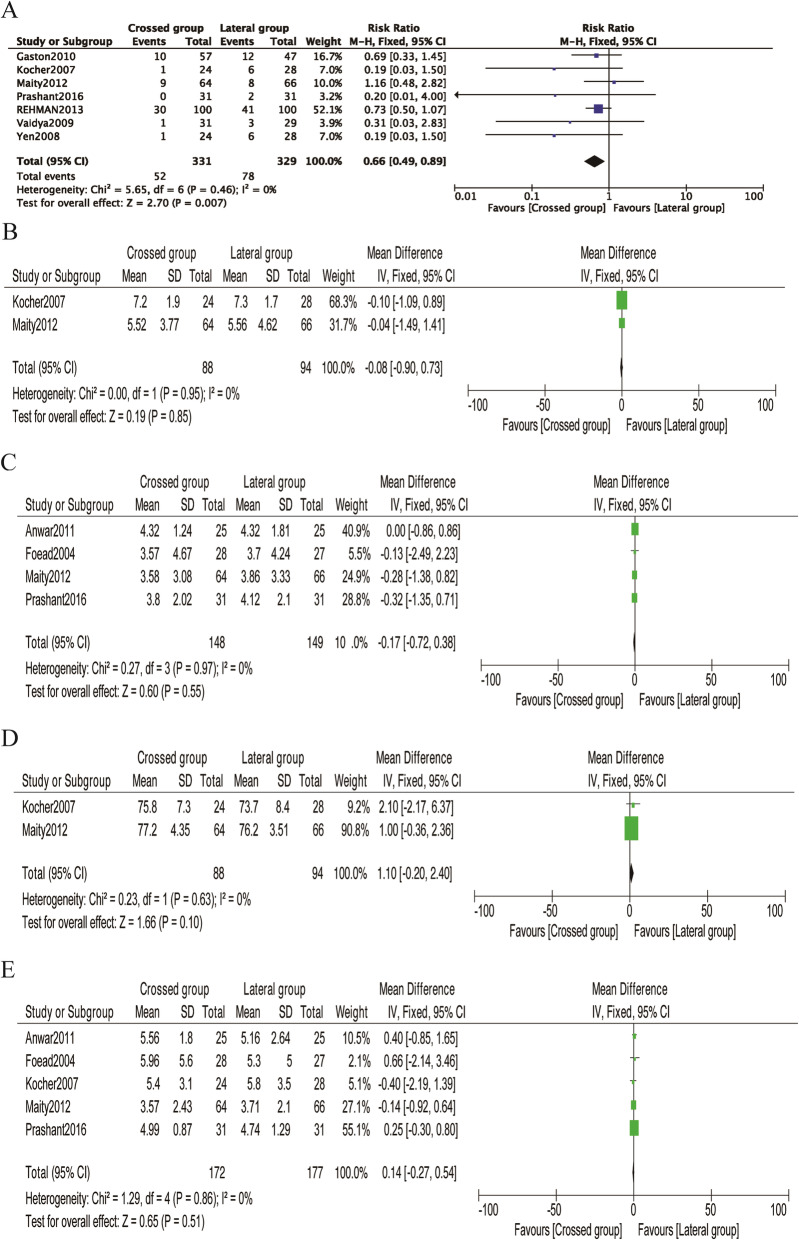
Comparison of radiographic outcomes between the lateral entry group and the crossed entry group. **A** Loss of reduction. **B** Carrying angle. **C** Loss of carrying angle. **D** Baumann angle. **E** Change in the Baumann angle

Nine studies [8, 27, 29–31, 34, 35] investigated the relationship between the type of Kirshner wire (K-wire) fixation and radiographic outcomes, among which the loss of reduction was reported in seven studies [8, 30, 31, 33–36]. There was no heterogeneity between the studies (P = 0.46, *I*^2^ = 0%). Loss of reduction occurred in 52 (15.7%) of 331 patients treated with crossed pins and in 78 (23.7%) of 329 patients treated with lateral pins. The pooled results showed significant differences between the two configurations (RR = 0.66; 95% CI 0.49–0.89, P < 0.05) (Fig. 7A). Crossed pins had an acceptable result compared to lateral pins. Publication bias was not significant (P = 0.251) (Figs. 5B and 6B).

Other evaluated measures of radiographic outcomes, such as the carrying angle, loss of carrying angle, Baumann angle, and change in the Baumann angle, also showed no significant differences between the crossed pin and lateral pin entry configurations (Fig. 7B–E, Table 2). The publication bias of change in the Baumann angle was not significant (P = 0.359) (Figs. 5C and 6C). Due to the limited number of included studied, publication bias analysis had not been done in the carrying angle, loss of carrying angle, and Baumann angle.

##### Functional outcomes

**Fig. 8:**
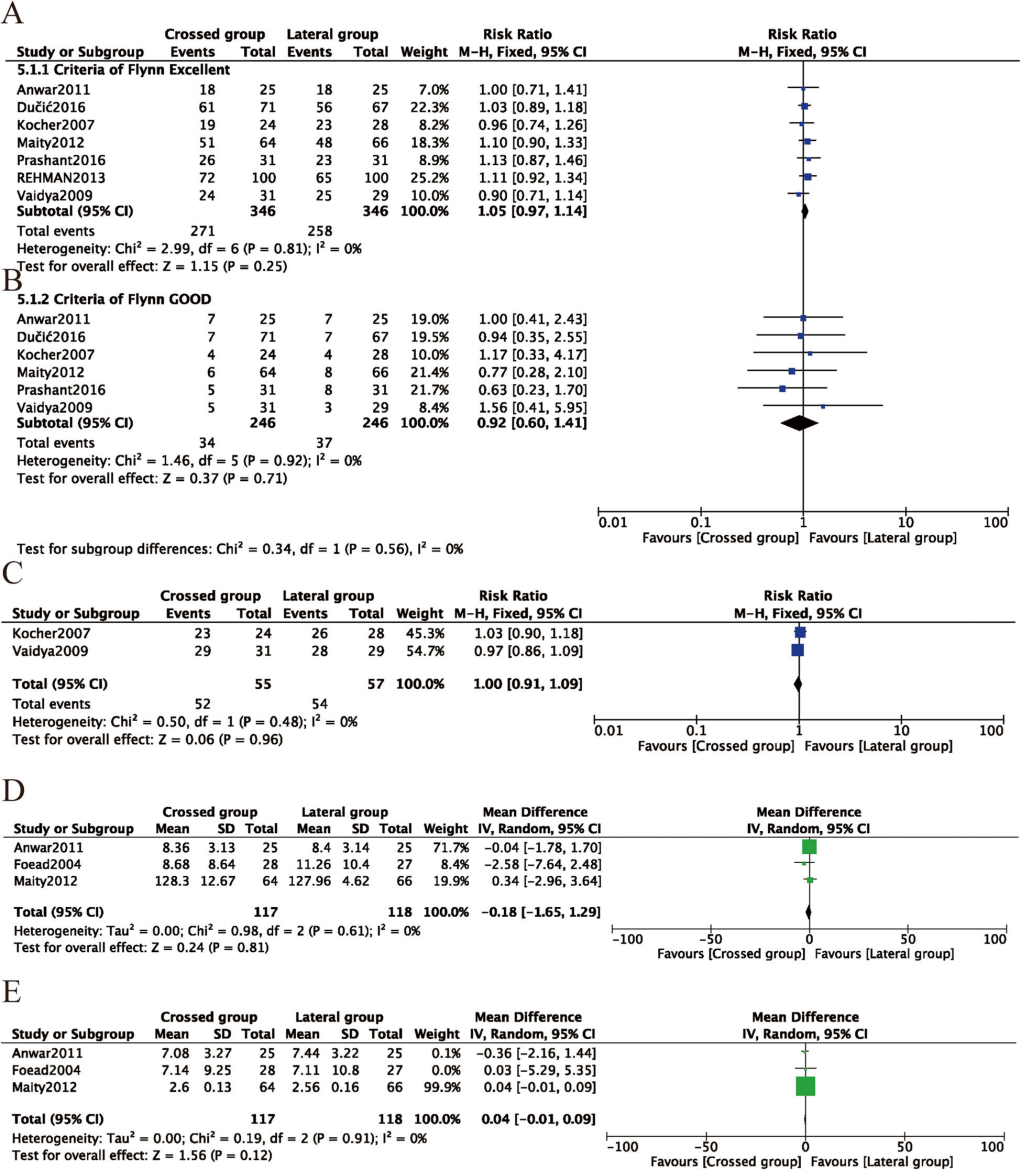
Comparison of functional outcomes between the lateral entry group and the crossed entry group. **A** Excellent Flynn criteria scores. **B** Good Flynn criteria scores. **C** Return to full function. **D** Loss of elbow flexion. **E** Loss of elbow extension

Flynn criteria scores for the functional and cosmetic outcomes were reported in seven studies [8, 27, 28, 31, 33, 35, 36]. Excellent outcomes were reported in 271 (78.3%) of 346 patients treated with crossed pins and 258 (74.6%) of 356 patients treated with lateral pins. According to the number of excellent and good Flynn scores, there was no difference in outcomes between the patients treated with crossed or lateral pin fixation (RR = 1.05, 95% CI 0.97–1.14, P = 0.25; RR = 0.92, 95% CI 0.60–1.41, P = 0.71) (Fig. 8A, B). The data were pooled for studies evaluating the total range of motion and flexion and extension, and no significant differences were found between the two fixation techniques (Table 2). Return to full function was reported in two studies. There was no significant difference between the two fixation methods (RR = 1.00, 95% CI 0.91–1.09, P = 0.96) (Fig. 8C). Loss of elbow flexion and extension was reported in three studies, and no difference was found between the two fixation methods (MD = −0.18, 95% CI −1.65–1.29, P = 0.81; MD = 0.04, 95% CI −0.01–0.09, P = 0.12) (Fig. 8D, E). Publication bias of the Flynn scores was not significant (P = 0.634; P = 0.206) (Figs. 5D, E and 6D, E).

#### Complications

**Fig. 9:**
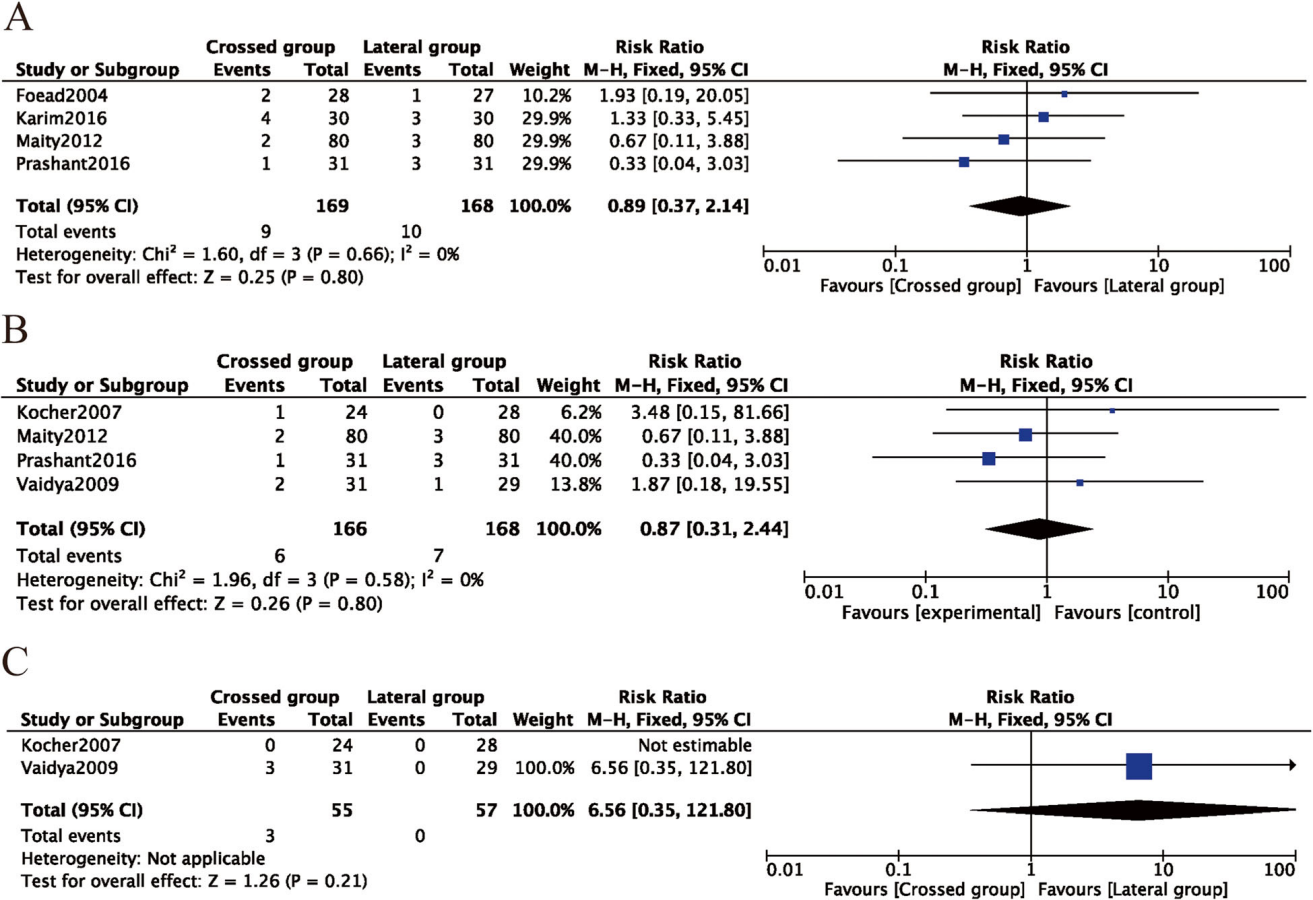
Comparison of complications between the lateral entry group and the crossed entry group. **A** Pin tract infection. **B** Superficial infection. **C** Reoperation

Four studies [26, 29, 31, 33] reported the incidence of pin infections. In the crossed pins group, nine (5.3%) of 169 patients had infections, whereas 10 (6.0%) of 168 patients in the lateral entry group had infections. There was no difference between the two fixation methods (RR = 0.89, 95% CI 0.37–2.14, P = 0.80) (Fig. 9A). Superficial infection was reported in four studies [8, 31, 33, 36], in which six (3.6%) of 166 patients were treated with crossed pins and seven (4.2%) of 168 patients were treated with lateral pins developed infections. There was no statistically significant difference between the two fixation methods in terms of superficial infections (RR = 0.87, 95% CI, 0.31–2.44, P = 0.80) (Fig. 9B). Heterogeneity analysis suggested that no statistical heterogeneity existed in the studies reporting pin infections and superficial infections (P = 0.66; *I*^2^ = 0%; P = 0.58; *I*^2^ = 0%). Two studies [8, 33] reported the incidence of reoperation, and three (3.6%) of 55 patients treated with crossed pins had infections, whereas none of the 168 patients in the lateral pin group had infections. There was also no difference between the two fixation methods (RR = 6.56, 95% CI 0.35–121.80, P = 0.21) (Fig. 9C). No evidence of publication bias was observed in studies reporting complications(P = 0.690; P = 0.127; P = 637) (Figs. 5F, G and 6F, G).

## Discussion

SCHF is the most frequent fracture of the elbow injury and often occurs in pediatrics aged 2–10 years [37]. They complain of continuous pain and crying regularly. The clinical presentation is swelling elbow and forearm, as well as a functional limitation of the up-limb elbow in the flexion and the forearm in semi-pronation. SCHF should be considered any injury even with a low energy mechanism, and lateral and oblique series X-rays are the cornerstone in the diagnosis of pediatric fractures. Diagnosis at the hospital was based on clinical examination and X-rays [38]. The study describes many methods of positioning the pinning fixation for the treatment of SCHF. The crossed pinning fixation has been demonstrated to be more reliable biomechanical stability [39], while the method leads to an increased risk of iatrogenic damage to the ulnar nerve versus lateral pinning fixation [17]. Because lateral pinning fixation has the risk of reduced stability, it is often necessary to insert more lateral pins to increase stability [40].

Our meta-analysis suggested that children with SCHF undergoing lateral pinning fixation had low rates of iatrogenic ulnar nerve injuries. However, the loss of reduction rate was lower in the crossed pin fixation group. The Baumann angle, carrying angle, change in Baumann angle, Flynn criteria scores, return to full function, loss of carrying angle, loss of elbow extension, loss of elbow flexion, pin tract infections, and superficial infections were not significantly different between the two treatment groups. In the subgroup, crossed pins with mini-open incisions had the same risk of iatrogenic ulnar nerve injury as lateral pins. These results support the use of crossed pinning entry with a mini-open incision as a therapeutic strategy that could improve the management of supracondylar fractures in children.

Some meta-analyses had reported decision-making for SCHF treatment in children. Zhao et al. [17] published a meta-analysis of cohort studies comparing surgical treatments for SCHF. In this article, the authors focused on comparing the clinical outcomes with different K-wire entries (lateral entry or crossed medial and lateral pinning techniques). The results suggested that crossed pinning fixation had a higher risk for iatrogenic ulnar nerve injury than the lateral pinning technique. Other outcomes including the carrying angle, loss of carrying angle, Baumann angle, change in Baumann angle, or the loss of reduction were not significantly different between the two surgical techniques. Woratanarat et al. [22] also demonstrated that cross-pinning minimized the risk of loss of fixation but increased the risk of ulnar nerve damage. Two fewer cases of loss of fixation occurred in the cross-pinning group compared to the lateral pinning group, but five more cases of ulnar nerve injury occurred. Hence, lateral pinning has been recommended. Na et al. [21] showed that the rate of ulnar nerve injury in the crossed group was significantly higher than that in the lateral entry group. The lateral entry technique was recommended for the treatment of SCHF.

Our results differed from the results reported in other meta-analyses, and our subgroup analysis showed that there was no difference in the risk of iatrogenic ulnar nerve injury between crossed pinning entry with mini-open incision and lateral needle insertion. At the same time, crossed pinning entry with mini-open incision had the same reliable biomechanical stability as crossed pinning entry. Therefore, our results concluded that the crossed pinning entry with mini-open incision technique could reduce the loss of reduction risk. Moreover, the risk of iatrogenic ulnar nerve injury was lower than in the lateral pinning entry group. The crossed pinning entry with mini-open incision technique is an effective therapeutic strategy for managing displaced supracondylar humeral fractures in children. Moreover, randomized controlled trials (RCTs) had the highest evidence level. As a powerful tool, meta-analysis utilizing RCTs could provide more reliable results than a single study by combining all eligible studies, especially in explaining controversial conclusions.

The crossed entry technique has a biomechanical advantage for displaced type II and type III supracondylar fractures [41, 42]. This method emphasizes the relative stability principle in supracondylar fractures and has gained popularity for its potential advantage of less loss of reduction [40]. However, the crossed entry fixation method has been shown to have a higher risk of iatrogenic ulnar nerve injury than the lateral entry method [43]. Some studies reported that the risk of iatrogenic ulnar nerve injury could be greatly reduced through the placement of a medial pin with a medial mini-incision on the medial epicondyle and the extension of the elbow [8, 26, 31, 33]. The risk of iatrogenic ulnar nerve injury associated with medial pin entry could be resolved after wound exploration and placement of the medial pin at a new location.

Our results suggested that there were significant differences in the number of iatrogenic ulnar nerve injuries and the reduction of function between children treated with crossed entry and lateral entry. Crossed pinning entry, consisting of crossed pinning entry and mini-open incision, did not significantly decrease the postoperative rate of complications, including pin infections, superficial infections, and the rate of reoperation, compared to the lateral pinning entry method. A total of 12 RCTs were finally identified for inclusion in this study. The quality of the studies, generally, was poor. The majority of the RCTs had insufficient information on the randomization methods. Only three of the included studies used sealed envelopes for allocation concealment. Blinding was reported in all studies, which showed a low risk of performance bias or detection bias.

Our study had several strengths. Firstly, this study was the latest meta-analysis of RCTs evaluating the effect of the two fixation methods for displaced supracondylar fractures. Although several related meta-analyses have been published, this meta-analysis included more RCTs through a more extensive search. Secondly, all of the included studies used a randomized controlled design, thus increasing the comparability between the two groups and reducing selection bias. Thirdly, literature searches were conducted in the Cochrane Library, PubMed, EMBASE, Web of Science, the Chinese Biomedical Literature Database, the China Journal Full-text Database, and the VIP Database.

However, several limitations should be acknowledged. Firstly, although a comprehensive literature search was conducted, some unpublished trials might have been missed, which would lead to non-publication bias. The authors of all 12 studies were contacted to ask for additional methodological and statistical information, but no responses were received. Secondly, the small number of included studies and the relatively small number of participants in each study restricted the statistical power. Thirdly, the lack of high-quality studies prevented us from investigating the heterogeneity of the studies. Clinical heterogeneity might be caused by preexisting conditions in the children, the experience level of the orthopedic surgeons, the fracture type, medical commodities, and follow-up time. The above confounding factors might have an impact on the study outcomes.

## Conclusions

Compared with lateral pinning entry, crossed pinning entry had a higher risk of iatrogenic ulnar nerve injury and increased structure instability. However, in the subgroups, crossed pinning with mini operative reduction decreased the risk of iatrogenic ulnar nerve injury. Therefore, the recommended strategy for the treatment of pediatric SCHF is crossed pinning entry with a mini-open incision, which can provide a stable elbow and avoid iatrogenic injury of the ulnar nerve.

## Data Availability

All data are fully available without restriction.
